# Sexual Experience Enhances *Drosophila melanogaster* Male Mating Behavior and Success

**DOI:** 10.1371/journal.pone.0096639

**Published:** 2014-05-07

**Authors:** Sehresh Saleem, Patrick H. Ruggles, Wiley K. Abbott, Ginger E. Carney

**Affiliations:** Department of Biology, Texas A&M University, College Station, Texas, United States of America; CNRS, France

## Abstract

Competition for mates is a wide-spread phenomenon affecting individual reproductive success. The ability of animals to adjust their behaviors in response to changing social environment is important and well documented. *Drosophila melanogaster* males compete with one another for matings with females and modify their reproductive behaviors based on prior social interactions. However, it remains to be determined how male social experience that culminates in mating with a female impacts subsequent male reproductive behaviors and mating success. Here we show that sexual experience enhances future mating success. Previously mated *D. melanogaster* males adjust their courtship behaviors and out-compete sexually inexperienced males for copulations. Interestingly, courtship experience alone is not sufficient in providing this competitive advantage, indicating that copulation plays a role in reinforcing this social learning. We also show that females use their sense of hearing to preferentially mate with experienced males when given a choice. Our results demonstrate the ability of previously mated males to learn from their positive sexual experiences and adjust their behaviors to gain a mating advantage. These experienced-based changes in behavior reveal strategies that animals likely use to increase their fecundity in natural competitive environments.

## Introduction

Animals use contextual information to determine how to behave in a particular situation, and behavioral adaptability is key in facing rapidly changing environments. Innate behaviors in animals are continuously affected by varying factors including, but not limited to, environment, physiological state, or experience [1], [2], [3], [4], [5], [6]. Within a population animals vary in their social experiences, including the number of times they have mated, and behavioral adaptations based upon sexual experience carry the potential to increase mating opportunities for more experienced animals. Optimizing strategies that increase mating success is particularly important, and prior sexual experiences as well as the current social environment potentially affect an animal’s strategy for obtaining mates. In mammals oxytocin promotes a variety of social behaviors, including sexual behavior, and sexually experienced male rats have higher levels of brain oxytocin receptors as well as shorter copulation latencies compared to naïve males [7]. Prior exposure to opposite sex pheromones also can change an animal’s olfactory sensory threshold [8], [9], which may allow more rapid mate detection and increase the probability of mating success. A social environment in which there is competition for mates can have different effects on mate choice depending upon the circumstances [2], and learning via social interactions has the potential to affect sexual selection and speciation [10]. Understanding the fundamental interactions between genotype and environment and their combined effect on phenotype is essential to understanding how evolutionary pressures shape various phenotypes, including behaviors.

*D. melanogaster* exhibit extensive behavioral plasticity, and the ability to genetically manipulate the fly makes *Drosophila* a very attractive model to study behaviors and their underlying genetics [11], [12], [13]. Like other animals, fruit flies have complex behavioral repertoires and sensory systems that inform the decision making processes for behaviors including egg laying [14], [15], [16] and mate choice [17], [18]. To woo a female, a *D. melanogaster* male performs a stereotypical suite of courtship behaviors, including following, orientation towards the female, tapping, unilateral wing extension and vibration, and licking, which ultimately culminate in mounting for copulation [17]. The potential for these elaborate male courtship behaviors is set genetically through the actions of male-specific protein products of the *fruitless* (*fru*) and *doublesex* (*dsx*) genes [18], and individual steps in the courting process occur in a precise order.

Although reproductive behaviors are genetically programmed and are performed by socially naïve individuals, particular aspects of these behaviors are plastic and modified by experience. Male flies inherently perform courtship towards a variety of potential mates, but social experience with same-sex or opposite-sex individuals changes subsequent male reproductive behaviors [19], [20], [21]. Males reared in male-dense environments during early adulthood copulate longer with females and have enhanced fecundity and fertility [21], and sexually immature males that have been courted by mature males are more sexually aggressive during courtship [22]. Males also learn short-term avoidance of non-receptive individuals [23], [24], [25]. For example, males that unsuccessfully court non-receptive mated females decrease later courtship efforts towards receptive females [23], a learning process known as courtship conditioning. This learned response was recently linked to an enhanced sensitivity to the lipid 11-*cis*-vaccenyl acetate (cVA), a component of the male ejaculate that is transferred to mated females, and the response to cVA is modulated by dopaminergic neuron signaling [26]. Surprisingly, males that have courted non-receptive females begin courting virgin females more rapidly [19]. *Drosophila* males also selectively change their behavior based on the nature of their prior sexual experiences. For instance, *D. melanogaster* males experienced at courting non-receptive, heterospecific *Drosophila simulans* females suppress courtship toward other *D. simulans* females but not toward receptive *D. melanogaster* females [27]. This learned courtship suppression occurs rapidly since male *D. melanogaster* reduce their courtship efforts towards *D. simulans* within 5 min of female exposure [28].

Most of the previously described paradigms investigated behavioral effects of social experiences that did not culminate in mating, but little attention has been directed to understanding how the presumably positive experience of mating affects later courtship behaviors and copulation encounters. Previously mated *D. mercatorum* males have shorter courtship latencies, and the amount of time a male spends courting increases between the first and second matings [29]. In contrast, Kujtan and Dukas [30] demonstrated that *D. persimilis* males that have mated with a heterospecific female do not have greater heterospecific mating success or altered courtship compared to naïve males or males that previously mated with conspecifics. A more recent study shows males that mate with conspecific females subsequently decrease courtship towards heterospecifics [31]. However, a detailed analysis of male courtship behavior and mating propensity towards conspecifics after a successful bout of mating is still lacking. Since males show behavioral modification based on their current and previous social experiences, including changes in courtship effort that may provide a mating advantage [29], we hypothesized that *D. melanogaster* males with prior mating experience would modify their courtship behaviors towards receptive females. One possibility is that sexually experienced males may have decreased mating latencies. To identify potential changes in behavior, we examined overall courtship effort of males and then quantified specific parameters of courtship performance. Since matings are highly competitive in the wild, we also asked if sexual experience provides males with a competitive advantage against males that lack such experience.

## Materials and Methods

### Fly Husbandry

All strains were maintained on standard cornmeal and sugar media in a 25°C incubator with 12 hr light/dark cycles. *Canton-S* wild-type flies that were backcrossed for 10 generations were used in all assays. Flies were sexed within 2 hrs of eclosion. Males were aged individually in food vials, while females were aged in groups of 10–15. All assays were carried out on 5 day old sexually mature flies and were recorded using JVC-HDD Everio cameras. Behaviors were analyzed by at least two researchers to avoid bias. All observations were conducted by observers blind to the treatment. Anesthesia was avoided on the day of the behavioral assays and flies were aspirated from one chamber to another. Courtship assays were carried out in 0.785 cm^3^ chambers with wetted filter papers. For every experiment described below, assays were performed over multiple days, and control and experimental animals were tested on the same day.

### Single Pair Mating Assays

One sexually naïve male was placed in a courtship chamber with one virgin receptive female and the pair was video recorded until the completion of mating so that male behaviors could be evaluated. To obtain a sexually experienced male, a 5 day old sexually naïve male was mated to a virgin female followed by a 30–45 min recuperation time at 25°C. This recuperation time was selected because in other learning paradigms where males demonstrated behavioral modifications due to social experiences, changes in behavior were quite rapid, beginning as early as 2 min after the training period, and lasting up to 24 hrs [19], [27], [32], [33]. The experienced male was then transferred to a new mating chamber with a virgin female and the pair was videoed until the completion of mating.

Courtship index (CI) is a common measure of a male fly’s sexual enthusiasm towards a female. Throughout this study, CI was calculated as the proportion of time a male spent courting (orientation, following, wing vibrations and abdomen bends) relative to the mating latency. Frequency and percent duration of wing extensions performed towards the female were calculated relative to the total male courting time. Abdomen bends included partial to full abdomen curvature when the male was oriented behind the female. Frequency of abdomen bends was calculated by recording the number of abdomen bends performed by the male and standardizing to courting time (*N* = 15–38).

### Competitive Mating Assays

One naïve and one sexually experienced fly were introduced into a courtship chamber followed by a virgin female. Males were distinguished by wing markings randomized between the two different types of males throughout the assays. Marking flies did not affect males in the competition assay as there was no significant difference in mating success between marked and unmarked males (Chi-square (1, *N* = 49)  = 1.00, *P* = 0.3173). Behaviors were recorded for 2 hrs or until completion of a mating by the winning male. A CI for each male in the assay was calculated relative to the latency to copulation of the winner. Individual courtship behaviors were measured as described above.

As a measure of male-male aggressive behavior during the competitive mating assays, we quantified the number of lunges, instances of males rearing up on their back legs followed by attacking an opponent with the forelegs [34], [35], performed by each male prior to initiation of copulation by the winning male.

### Female Preference in Competitive Mating Assays

The contribution of different sensory systems to female preference in a competitive assay was measured by ridding the virgin female of various sensory modalities that are involved in mate selection. Females were either blinded by application of black acrylic paint over their eyes a day prior to testing, or competitive assays were run in red light. Deaf females were obtained by surgically removing aristae [36]. Removing the aristae together with the 3^rd^ antennal segment reduces both hearing and olfaction [37].

### Courtship Experienced or Incompletely Mated Rivals

To obtain a courtship experienced male fly, we allowed a naïve male to court a non-mateable sexually mature virgin female for 13 min, which was determined to be the average mating latency for naïve males (*N* = 85). Non-mateable females were produced by super gluing their genitalia 24 hrs prior to the assay. After training with the female, the courtship experienced male was isolated in a food vial and allowed to recuperate for 30–45 min at 25°C. After the rest period, a courtship experienced male was competed against a naïve male for a mating with a virgin female as described above. The winner of each competition assay was noted. Individual courtship behaviors were measured as described above (*N* = 40–42).

In a separate assay, males were allowed to complete courtship by mounting the female, but the copulation was interrupted within the first 30–45 sec when the pair was separated by gentle tapping on the courtship chambers. The male then recuperated for 30–45 min at 25°C. An incompletely mated male was then placed in a competition assay with a naïve male and the winner of each competition assay was noted. We examined fertility of 21 of the mated females and found that only 14% sired any progeny, indicating there was little sperm transfer during this period.

### Statistical Analyses

JMP Pro 11.0.0 software was used for all statistical analyses. Courtship parameter data were arcsine-transformed and checked for normality (Shapiro-Wilk test) and homoscedasticity (Brown-Forsythe or Levene tests). Due to the non-normal distribution of the data, significant differences in individual courtship parameters between experienced and naïve males in single pair or competitive mating assays were tested using the Wilcoxon test. We also used the Wilcoxon test to assess differences in courtship behaviors of the same fly before and after gaining sexual experience and to determine differences in aggression levels between naïve and experienced males in competitive mating assays. Figures display mean ± s.e.m. values for CI, wing extension frequency and duration, and abdomen bend frequency.

## Results

### Sexual Experience and Behavior

**Figure 1:**
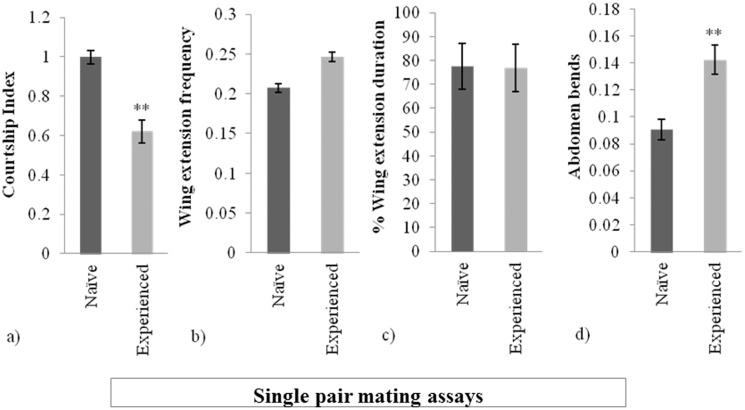
Sexually experienced males behave differently than naïve males towards receptive females. (a) courtship index, (b) wing extension frequency, (c) wing extension duration, and (d) abdomen bend (copulation attempt) frequency, for individual males in single pair assays (one male plus a receptive female). **p<0.001. Error bars denote mean ± s.e.m. values.

We examined the effects of prior mating experience on subsequent male behaviors by asking whether *D. melanogaster* males that had mated once previously (referred to here as sexually experienced males) alter their courtship and mating behaviors towards receptive females. We determined courtship performance of individual sexually experienced or naïve males that were placed with a receptive female (single pair mating assays). First, we calculated a composite behavioral index, the courtship index (CI), which reflects a male fly’s overall courtship efforts. In single pair assays, sexually experienced males spent significantly less time courting than sexually naive males (Fig. 1a) but had reduced mating latencies (Mating latency: naïve = 947±105.15 sec, *N* = 36, experienced = 732.54±95.31 sec, *N* = 28, Wilcoxon signed-rank test, Z = −2.117, *P* = 0.0342); there was no effect of sexual experience on time to courtship initiation (courtship latency) or copulation duration (values indicate mean ± s.e.m. Courtship latency: naïve = 122.31±92.63 sec, *N* = 36, experienced = 145.43±190.63 sec, *N* = 28, Wilcoxon signed-rank test, Z = −0.409, *P* = 0.8763; Copulation duration: naïve = 1427±35.31 sec, *N* = 36, experienced = 1484±68.68 sec, *N* = 28, Wilcoxon signed-rank test, Z = 0.3789, *P* = 0.7047).

Since sexually experienced males had lower CIs but decreased time to mating, we determined if these males differed from naïve males in the performance of component courtship behaviors. In single pair assays sexually experienced males had higher abdomen bend (copulation attempt) frequencies (Fig. 1d). We detected a non-significant trend towards increased wing extension frequency by experienced males, but wing extension duration did not differ between the two types of males in single pair assays. To confirm that males change their behavior as a consequence of sexual experience, we also compared the behaviors of a male both before and after he gained sexual experience. Individual males spent less time courting a virgin female after gaining sexual experience (CI: naïve = 1.036±0.030, experienced = 0.632±0.093, *N* = 27, Wilcoxon signed-rank test, Z = 4.428, *P*<0.0001), and they increased their efforts in other courtship parameters (Wing extension frequency: naïve = 0.237±0.005, experienced = 0.308±0.004, *N* = 12, Wilcoxon signed-rank test, Z = −2.338, *P* = 0.0194; Wing extension duration: naïve = 100±0.04, experienced = 99.01±0.017, *N* = 12, Wilcoxon signed-rank test, Z = 0.259, *P* = 0.795; Abdomen bend frequency: naïve = 0.0861±0.005, experienced = 0.141±0.003, *N* = 26, Wilcoxon signed-rank test, Z = −3.642, *P* = 0.0003).

**Figure 2:**
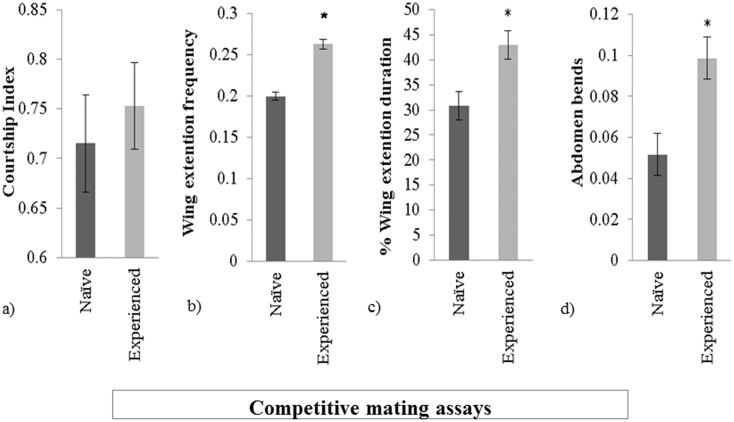
Sexually experienced males out-perform naive rivals. In competitive assays, the behaviors of each male were quantified. (a) courtship index, (b) wing extension frequency, (c) wing extension duration, and (d) abdomen bend frequency for each male in a competitive mating assay (two males plus a receptive female). *p<0.01. Error bars denote mean ± s.e.m. values.

In the second set of experiments, we assessed behaviors of a sexually experienced male and a naïve male that were placed together in a courtship chamber with a receptive female (competitive mating assays). In contrast to single pair assays, when males were in direct competition for a mating, sexually experienced and naïve males performed similar overall levels of courtship (Fig. 2a, Wilcoxon signed-rank test, Z = −0.863, *P* = 0.3877). The CIs of naïve males were reduced in the competitive mating assays, while sexually experienced male CIs were not affected by the assay type (Fig. 1a and 2a). However, in competition assays, sexually experienced males outperformed naïve males in each of the three component behaviors (Fig. 2b–d). Although there are significant differences in wing extension duration, both types of males spend less time extending their wings in competitive mating assays, an effect that was detected previously [38].

One possibility is that sexually experienced males are initially more attractive or better at mating. To address this possibility, we quantified individual mating success rates for sexually naïve and sexually experienced males. Flies that did not mate within 2 hrs were discarded. Approximately 15% of animals tested (naïve as well as experienced) fell into this category. Therefore, 85% of experienced or naïve males successfully mated, and similar rates were detected throughout our experiments, indicating there was no significant difference in the mating success of naïve or sexually experienced males.

The increases in wing extension and abdomen bending frequency in sexually experienced males suggested to us that experienced males were more sexually aggressive. We wondered if this aggression was limited to sexual behavior or if experienced males were generally more aggressive. As a measure of aggression we quantified the number of lunges performed by each male towards the other male prior to copulation in competitive mating assays. Naïve and sexually experienced males did not differ in the frequency of aggressive lunges towards each other (lunges: naïve = 2.07±2.75, experienced = 2.02±2.39, *N* = 42, Wilcoxon signed-rank test, Z = −0.24239, *P* = 0.8085).

### Copulation Confers a Competitive Advantage to Sexually Experienced Males

**Figure 3:**
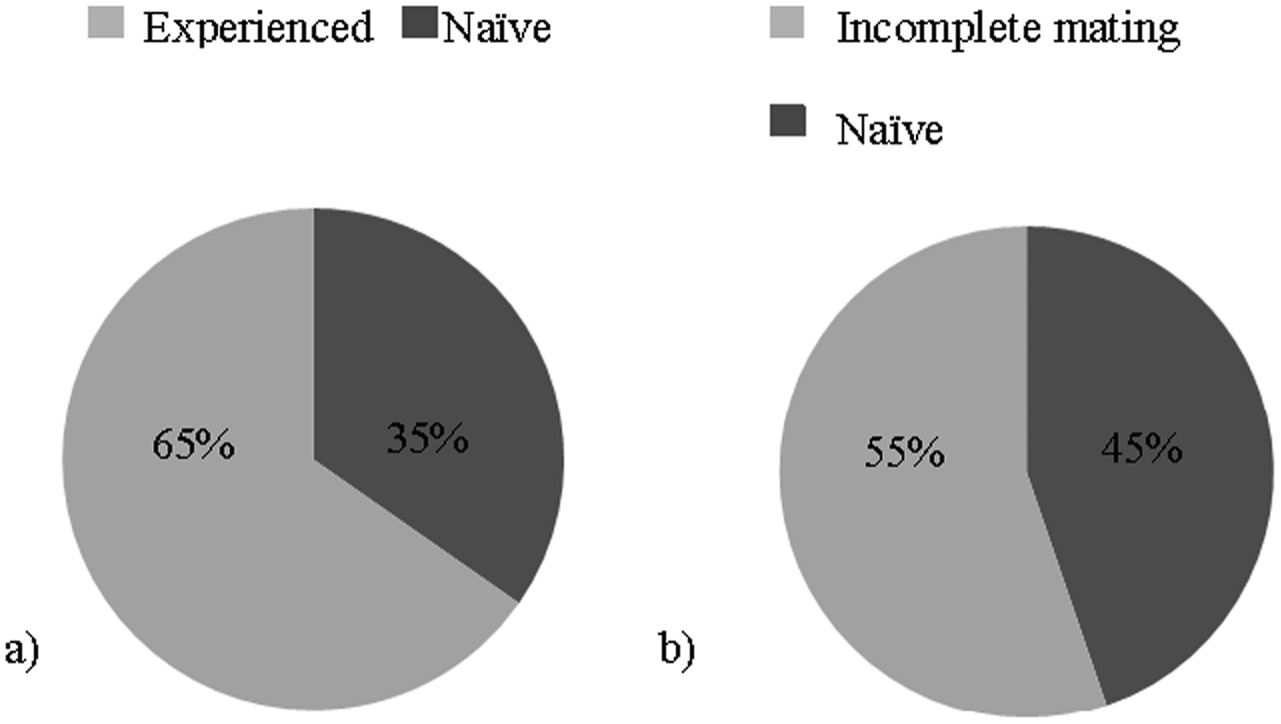
Sexually experienced males out-compete naïve rivals for matings. (a) Sexually experienced males (those that were courtship experienced and completed mating) out-competed naïve rivals for matings with receptive females. (b) Courtship experience followed by an incomplete mating did not provide a competitive advantage to sexually experienced males.

To assess whether sexual experience provided males with a competitive advantage in acquiring a mate, we determined which male achieved the mating in competitive mating assays. Males with prior mating experience achieved significantly more matings than naïve males when competing for females (Chi-square (1, *N* = 49)  = 4.592, *P* = 0.032.) (Fig. 3a).

Mated males are experienced in both courtship and the act of copulation. To identify the aspect of sexual experience that is important in providing a competitive mating advantage, we asked whether courtship experience alone was sufficient to provide this advantage. We restricted experience to only courtship by allowing a naïve male to court a non-mateable virgin female whose genitalia had been glued to prevent intromission. These courtship experienced males were then competed against naïve males for mating. Courtship experience with glued females was not sufficient in providing a mating advantage since there was not a significant effect on the number of matings (courtship experienced = 37%, naïve = 63%, Chi-square (1, *N* = 30)  = 2.133, *P* = 0.144). In order to reduce a possible negative association from courtship experience with glued females due to their inability to mate, we allowed males to court and copulate with non-glued receptive females but gently interrupted the matings within 30–45 sec to ameliorate effects of mating. Neither overall courtship nor any of the individually evaluated behaviors of these courtship experienced males were different from those of naïve male competitors (data not shown), and males that successfully courted but had incomplete matings did not have a competitive mating advantage against naïve males (Chi-square (1, *N* = 38)  = 0.421, *P* = 0.516) (Fig. 3b).

### Female Choice between Competing Males

Since sexually experienced males changed their courting behavior and were more successful in competing for mates, we wanted to identify the sensory modalities females used to distinguish between males of varying experience. Therefore, we selectively abrogated female sensory systems to determine the effect on competitive mating. We first tested for an effect of eyesight on female mate choice using two common paradigms to reduce female vision. We either covered both eyes of the female with black paint or performed the competitive mating experiments in dim red light. When black paint was applied to the eyes of focal females, sexually experienced males won significantly more matings (sexually experienced = 70%, naïve = 30%, Chi-square (1, *N* = 40)  = 6.4, *P* = 0.0114). When competitive assays were carried out in red light, a situation in which individuals of both sexes have reduced vision, there was no effect of sexual experience on mating success (sexually experienced = 54.7%, naïve = 45.3%, Chi-square (1, *N* = 42)  = 0.381, *P* = 0.5371).

Naïve and experienced males did not differ significantly in attaining matings when the focal female was deafened by removal of aristae (sexually experienced = 60.5%, naïve = 39.5%, Chi-square (1, *N* = 43)  = 1.884, *P* = 0.1699) or when both aristae and the 3^rd^ antennal segments were removed from the females to reduce their ability to detect olfactory as well as auditory signals (sexually experienced = 57.3%, naïve = 42.7%, Chi-square (1, *N* = 38)  = 0.105, *P* = 0.7456).

## Discussion

Our understanding of how and why fruit flies modify their behavior after exposure to various sexual encounters has come a long way since the seminal study by Siegel and Hall [23] demonstrating courtship learning in *D. melanogaster*. The results of our study demonstrate for the first time that a successful conspecific mating experience enhances a *D. melanogaster* male’s ability to compete for new mates, and we show that the male’s success is linked to changes in courtship behavior. Ability to survive and successfully face changing environments is often accompanied by variation in behavioral tactics [39], [40], [41], and such changes in behavior due to experience meet common definitions of learning [42], [43]. Sexual experience provides animals the opportunity to hone their skills and improve courtship towards future mates, gaining advantage over naïve individuals who lack such experience.

### Signal Evaluation

*D. melanogaster* evaluate visual, tactile, olfactory, gustatory, and auditory information prior to choosing a mate. Males use olfactory and gustatory information to determine female sexual maturity and species identity [17], and males unable to smell during courtship have drastically reduced mating success [44]. However, no single sensory system is required for successful mating, and there is a complex interaction between the sensory systems that affects courtship and mating success [45]. Less is known about how the female makes her choice using the social/sexual information that she collects, but male cuticular hydrocarbons and song are strongly implicated [17], [46], [47]. The female’s willingness to mate is affected by her perception of male signals and is indicated by her decreased locomotory movement to allow the male to gain physical contact for copulation [17], [47].

We wondered what male attributes the females evaluate to make their choices since they show increased receptivity towards experienced males as evidenced by the shorter mating latencies in single pair matings. Experienced males change their courtship, particularly in relation to song performance and copulation attempts (Figs. 1 and 2). In both cases the female could be using visual cues as a means of identifying differences in male performance to inform her choice. The impact of vision on female mate choice in *Drosophila* has been demonstrated in the context of mate copying. *D. melanogaster* females given a choice between two virgin males preferentially mate with virgin males of the phenotype that they previously observed copulating rather than males of another phenotype that they saw being rejected [48]. Discrimination does not occur when females do not see which type of male mates, demonstrating that visual cues can influence female mate choice.

The females in our study did not watch the sexually experienced males mate and therefore did not have this type of public information available to aid in making their decisions. Instead, they assessed a suitor based upon traits that could be directly evaluated during the courtship interaction, including behavioral and chemical cues. We tested the contribution of eyesight to mating success when two novel males (one naïve and one sexually experienced) are presented to a virgin female who cannot see. Experienced males achieve significantly more matings than naïve males when only the female is blinded, suggesting that sight is not critical to females in distinguishing the males. Instead, females may rely more heavily on an alternate sensory modality such as audition, gustation or olfaction in their selection of a mate. In our red light study, which also removes sight as a potential recognition mechanism, there is no significant effect of experience on mating success. This observation appears to contradict our results with blinded females in white light conditions. However, neither males nor females are able to see in red light, and visual cues are important for male courtship and mating efficiency [45], so we are likely detecting an effect on mating due to the males being unable to see.

During wing vibration, *Drosophila* males produce an acoustic signal that functions in species recognition [49]. The song is composed of pulse and sine components, each of which plays a distinct role in mate choice [50], [51], [52]. Wingless males lacking the ability to emit acoustic signals are less likely to mate with females than their winged counterparts, demonstrating the importance of wing vibrations to mating success [53]. Given the importance of song to female mate choice [47], the female may be responding to changes in song production by experienced males. In support of this hypothesis, there is no significant effect of sexual experience on mating success with females lacking aristae, which renders them incapable of hearing. Similarly, females that can neither hear nor smell no longer prefer experienced males. Both sets of experiments implicate hearing as an important female determinate of experienced male mating success and are consistent with the observed changes in experienced male wing extension frequency and duration. However, our findings do not allow us to definitively rule out olfaction as a modality involved in female mate choice in the competitive mating assays.

Another likely influence in female mate choice is the male cuticular hydrocarbon bouquet. Females perceive the dominant male cuticular hydrocarbon, 7-tricosene (7T), as an attractant [54]. Less is known about how females respond to female-specific cuticular hydrocarbons, which may transferred to the male during mating [55], [56]. (A recent report contradicts these earlier studies on hydrocarbon transfer [57].) Most cuticular hydrocarbons are believed to be detected via gustatory receptors present on external appendages [37], [58], although the olfactory system also is implicated in 7T detection by females [54]. It is plausible that the female detects a difference in the hydrocarbon profile between experienced and naïve males via her gustatory system and considers this information when making her choice between the two males that are vying for the mating. Testing a role for gustation in choice requires genetic rather than physical manipulations since gustatory receptors are widely distributed across the insect body, including appendages such as legs [37], [58] that are vital for locomotion during mating interactions.

### Male Experience

To understand which aspect of sexual experience provides male flies with a competitive advantage, sexually mature males were allowed to court sexually mature virgin females that were incapable of mating because we glued their genitalia. In this assay courtship experience alone is not sufficient in providing males with a competitive advantage against naïve males. We noted a trend toward naïve males outcompeting males experienced only with courting, giving rise to the possibility that the courtship experienced males had a negative association between failure to mate and courtship. Other work has demonstrated that males perform lower levels of courtship after being rejected in their earlier courtship efforts toward non-receptive females [19] and a recent study confirmed that males perceive failure to mate as a negative experience [26]. In contrast, the sexually experienced males in our initial competitive mating assays had both a successful courtship and a successful mating. When we allow males to court and copulate briefly (30–45 sec) with receptive females, courtship experienced males still do not have a competitive advantage against naïve males (Fig. 3b) and do not behave differently than naïve males in any of the measured courtship behaviors. It remains possible that an incomplete mating is perceived negatively by the male and does not provide the same type of positive, reinforcing stimulus that is required to elicit a behavioral change. Despite this potential caveat, we conclude that a longer copulation period is important for providing sexually experienced males with a competitive advantage in subsequent matings. This advantage may be derived from mechanosensory stimulation of the male or may be associated with transfer of one or more ejaculate components. Since mating in *Drosophila* is always preceded by courtship, it is not possible to independently assess the contribution of copulation to this courtship learning process.

The changes in behavior of sexually experienced males also could be attributed to increased sexual arousal, which is generally defined by decreased courtship latency and increased CI towards the female [59] and an increase in erratic courtship performance [60]. It is unlikely that the sexually experienced males in our study are generally sexually aroused since these males have lower CIs than naïve males (Fig. 1a) and similar courtship latencies. Later steps in the courtship ritual may require higher activation thresholds [61], [62], so another possibility is that these thresholds are reduced in sexually experienced males. We consider this possibility unlikely since courtship and abdomen bend latencies do not differ significantly between naïve and experienced males. It appears that experienced males are not more sexually aroused, but have instead learned from their prior experience and are applying this newly learned knowledge in the next mating encounter. Such experience-dependent behavioral modification could be mediated by *dsx* [63].

The males we tested had only one prior mating, which is sufficient to provide a competitive advantage and improve courtship performance. In contrast, other studies found that younger males that had mated previously were less preferred by females, implying that female *Drosophila* sense that multiply-mated males have a depleted ejaculate [64], [65] or that males are less attractive as mates because they carry female-specific pheromones due to physical contact during copulation. Indeed, the presence of the female cuticular hydrocarbon, 7,11-heptacosadiene, on male cuticles leads to a dose-dependent reduction in male mating [66]. Our study appears to contradict these earlier results, but there are variations in the experimental design among the studies that could account for the observed behavioral differences, including age of the test flies (3 days old compared to 5 days in our study), rearing conditions, and the amount of time between trials. However we consider the most likely explanation to be the degree of male sexual experience, which may affect behavior as well as the male’s chemical profile. Interestingly, the number of prior male ejaculations affects female prairie vole mate choice, with naïve and singly mated males being equally preferred, whereas thrice mated males are much less preferred [67]. Taken together, the results from our work and those of Markow et al. [64] are consistent with the observations in the vole study system and support the idea that female mating preferences are affected by the extent of male mating experience. Our work suggests that sexual experience has a positive effect on male competitive ability, at least initially. However, females are also adapting to males they encounter and, after multiple matings by the male, female preference for males with less sexual experience appears to play a stronger role in determining male *Drosophila* mating success.

Since pheromonal signal detection is an important determinant in *Drosophila* mate choice [46], [68], a likely explanation for female aversion to multiply-mated males is that the males have an odor that is displeasing. Adult males have detectable cVA on the tip of the ejaculatory bulb [56], and a mating increases male cuticular cVA [69]. Both sexes respond to cVA via the Or67d receptor, with females finding cVA appealing in the context of mating whereas males respond negatively to cVA [70]. It is possible that multiple ejaculations further increase male cuticlar cVA levels, and that females find higher doses of cVA aversive in the context of the male cuticular bouquet. Alternatively, there may be other aversive signals that increase in concentration on males due to multiple matings or longer mating durations. One candidate is the female pheromone 7, 11-heptacosadiene, which is present on mated male cuticles [66] and may increase with multiple matings. Changes in concentration of either or both of these cues may serve females as an indirect measure of the amount of male ejaculate available to fertilize their eggs. Therefore, increasing their concentration as a consequence of multiple matings may abrogate any advantage the male has gained due to changes in courtship performance.

The observed changes in courtship strategy by sexually experienced males may provide them a mating advantage over sexually naïve males in natural competitive situations, thereby increasing the fecundity of sexually experienced males. An explanation for enhanced courtship performance by singly mated males is that the positive experience of copulation increases “sexual confidence” by decreasing male sensitivity to cVA [71]. However, the signaling mechanisms contributing to enhanced courtship performance remain to be determined as do the mechanisms underlying increased female aversiveness to multiply-mated males. Coupled with the behavioral paradigm described here, the availability of genetic tools makes it possible, as a next step, to identify the loci and brain regions, such as dopaminergic reward systems [72], [73], which modulate male behavioral changes associated with a positive sexual experience.
